# Antihepatocarcinoma Effect of* Portulaca oleracea* L. in Mice by PI3K/Akt/mTOR and Nrf2/HO-1/NF-*κ*B Pathway

**DOI:** 10.1155/2017/8231358

**Published:** 2017-06-04

**Authors:** Zheng Guoyin, Peng Hao, Li Min, Gu Wei, Chen Zhe, Ling Changquan

**Affiliations:** Department of Traditional Chinese Medicine, Changhai Hospital, Second Military Medical University, Shanghai, China

## Abstract

The purpose of the present study was to evaluate the pharmacological effects of* Portulaca oleracea* L. (Purslane) (PL) on N-nitrosodiethylamine- (NDEA-) induced hepatocellular carcinomas (HCC) and explore its potential mechanism. Mice were randomly assigned to four groups: control group, NDEA group, NDEA + Purslane (100 mg/kg) group, and NDEA + Purslane (200 mg/kg) group. The animal of each group was given NDEA (100 ppm) in drinking water. 1 h later, Purslane dissolved in PBS was intragastrically administered for continuous seven days. The results showed that Purslane reduced the activities of alanine aminotransferase (ALT) and aspartate aminotransferase (AST) in liver and serum. Purslane also reduced the contents of interleukin-6 (IL-6), IL-1*β*, tumor necrosis factor-*α* (TNF-*α*), and methane dicarboxylic aldehyde (MDA) and restored the activity of superoxygen dehydrogenises (SOD) in serum. Purslane could obviously attenuate the hepatic pathological alteration. Furthermore, treatment with Purslane effectively inhibited the phosphorylations of phosphatidylinositol 3 kinase (PI3K), protein kinase B (Akt), mammalian target of rapamycin (mTOR), nuclear factor-kappa B (NF-*κ*B), and inhibitor of NF-*κ*B*α* (I*κ*B*α*) and upregulated the expressions of NF-E2-related factor 2 (Nrf2) and heme oxygenase- (HO-) 1. In conclusion, our research suggested that Purslane exhibited protective effects on NDEA-induced hepatocellular carcinomas by anti-inflammatory and antioxidative properties via the PI3K/Akt/mTOR and Nrf2/HO-1/NF-*κ*B pathway.

## 1. Introduction

Hepatocellular carcinoma (HCC) is the sixth most commonly diagnosed cancer with high mortality in the world [1]. The median survival period of HCC is less than 12 months from diagnosis [2]. Several kinds of treatments might be beneficial for HCC, such as local ablative therapy, liver transplantation, resection, hepatic artery transcatheter treatment, and surgical therapy. However, limited effective chemotherapy agent available for HCC patients has been found. Thus, there is an urgent need to search for novel therapeutic strategies and agents [3–5].

The released proinflammatory cytokines recruit the inflammatory condition in the microenvironment for tumor development. Therefore, HCC is also acknowledged to be a result of an inflammatory accumulation of hepatocyte leading to cirrhosis [6]. In addition, biomolecule targets of oxidative stress conduce to nuclear fragmentation membrane disruption and lipid peroxidation which may trigger carcinogenesis [7]. Liver, the major organ which metabolizes ingested material, thus is more susceptible to carcinogenic insult. N-Nitrosodiethylamine (NDEA) is widespread in tobacco smoke, beverages, agricultural chemicals, and industrial pollution, which are the important risk factors of hepatic disorders [8]. The NDEA-induced hepatocarcinogenicity is a common screening model to evaluate the hepatoprotective compound with antioxidant properties.

Purslane (PL), a member of the Portulacaceae family, is an annual succulent herb distributed as turfgrass weed or field crop in diverse geographical environments throughout the world [9]. Purslane is one of the most useful medicinal plants and is named “Global Panacea” by the World Health Organization [10, 11]. Former research confirmed the higher nutritional quality of Purslane in most cultivated vegetables because of its higher levels of alpha-linolenic acid, ascorbic acid, and beta-carotene [10, 12]. As a Traditional Chinese Medicine, Purslane is now widely used for its various pharmacological effects including wound-healing, analgesic, antiaging [13], antioxidative [14], and anti-inflammatory [15] activities. Evidence has emerged indicating the hepatoprotective effect of Purslane on CCl_4_-induced liver damage and acetaminophen-induced liver injury through the anti-inflammatory and antioxidative properties [16, 17]. It is noteworthy that Purslane shows antitumor activity in human hepatocellular carcinoma cells (HepG2) [18]. However, whether Purslane exhibits protective effect on liver cancer in vivo remains limitedly elucidated. The present study was to evaluate the pharmacological effects on Purslane on N-nitrosodiethylamine- (NDEA-) induced hepatic oncogenesis in AKR mice and tried to investigated its potential mechanism.

## 2. Materials and Methods

### 2.1. Reagents

Purslane was purchased from the market in Yucheng, Henan province, China. N-Nitrosodiethylamine (NDEA) was supplied from Sigma-Aldrich. Mouse IL-1*β*, IL-6, and TNF-*α* enzyme-linked immunosorbent assay (ELISA) kits were produced by Nanjing KeyGen Biotech. Co., Ltd. SOD and MDA kits were provided by Nanjing Jiancheng Bioengineering Institute (Nanjing, China). All antibodies were obtained from Cell Signaling Technology Inc. (Beverly, MA, USA).

### 2.2. Preparation of Purslane Extract

Generally, Purslane was washed thoroughly with water and dried in the shade. The powdered Purslane (12 kg) was ground and extracted in ethanol (80 L) at room temperature for 7 d, and the filtered extract from ethanol was obtained under reduced pressure. The solvent was evaporated under vacuum to get rid of ethanol. Then a crude extract Purslane was achieved. Before the experimental use, Purslane extract was dissolved in dimethyl sulfoxide (DMSO) and the final concentration of DMSO was less than 0.05%.

### 2.3. Animals

40 Male AKR mice (6 to 8 weeks) were obtained from Jiangning Qinglongshan Animal Cultivation Farm (Nanjing, China). Animals had free access to water and food supplied in special steel containers. During the experiment period, mice were maintained in an air-conditioned room at 23 ± 2°C with a 12-hour light/dark cycle. All the experimental procedures were performed according to the National Institutes of Health Guidelines for the Care and Use of Laboratory Animals.

### 2.4. Experiment Protocol

Mice were randomly assigned to four groups: control group, NDEA group, NDEA + Purslane (100 mg/kg) group, and NDEA + Purslane  (200 mg/kg) group. The animal of each group was given NDEA (100 ppm) in drinking water. Simultaneously, mice in control group were treated with normal drinking water. 1 h later, Purslane (100 mg/kg, 200 mg/kg) dissolved in 0.5 ml PBS was intragastrically administered for continuous seven days. After the last treatment of Purslane, the mice were sacrificed. Blood were harvested from hearts and allowed to clot at laboratory temperature for 20 min and then centrifuged at 3000 rpm/min for 10 min to obtain serum. The serum samples were stored at −80°C for pending tests. Three of the liver tissues were excised for the histopathological observation. The other livers were collected and kept at −80°C for western blot analysis.

### 2.5. Determinations of Liver Functional Enzymes in Serum and Liver Tissues

Liver samples were homogenized with cold normal saline and then centrifuged at 12,000 rpm for 10 min at 4°C. Thereafter, the supernatant of the homogenate was harvested into tubes and maintained at −80°C. The protein contents were determined using a BCA protein assay kit.

The concentrations of ALT and AST in serum and liver tissues were determined using commercial kits according to the instructions recommended by the manufacturers (Nanjing Jiancheng Bioengineering Institute, Nanjing, China).

### 2.6. Measurements of TNF-*α*, IL-1*β*, and IL-6 in Serum and in Liver

The serum and liver concentrations of TNF-*α*, IL-1*β*, and IL-6 were measured by ELISA kit in accordance with the manufacturers. The optical density (OD) was read at 450 nm with a microplate spectrophotometer. Consequently, the contents were calculated according to the standard curves.

### 2.7. Determinations of Antioxidant System and Lipid Peroxidation Products in Serum

The operations for examination of SOD and MDA were conducted according to the instructions of commercial kits (Jiancheng Institute of Biotechnology, Nanjing, China).

### 2.8. Histopathological Examination

At the end of the experiment, liver tissues were dissected out from the left lobe of livers. The liver specimens were fixed in 10% neutral formalin and then embedded in paraffin blocks. The paraffin-embedded slices with 4 *μ*m sections were stained with hematoxylin and eosin (H&E) (Sigma, Korea) according to the standard procedures for photomicroscopic assessment (200x magnification). Finally, pathological changes in the hepatic tissues were observed in a blinded manner under a light microscope. Liver inflammatory cell count based on a five-point scoring system was performed to estimate the severity of leukocyte infiltration. The scoring system was as follows: 0: no cells, 1: a few cells, 2: a ring of cells with a thickness of 1 cell layer, 3: a ring of cells with a thickness of 2–4 cell layers, and 4: a ring of cells with a thickness of more than 4 cell layers.

### 2.9. Western Blot

Liver tissues were homogenized in lysis buffer. The concentrations of total protein were determined by a BCA protein assay (Beyotime, Nanjing, China). Equal protein extract was mixed with 5 times loading dye and 2-mercapto ethanol followed by heating at 95°C for 5 min. Afterwards, the samples were subjected to 10% SDS-PAGE gels and electrotransferred to a polyvinylidene difluoride membrane. The membrane was treated with 5% nonfat milk in 1x TBS with 0.25% Tween-20 (TBST) to block nonspecific antibody binding site and then was incubated with the appropriate concentration of specific antibody. After washing in TBST three times, the blot was incubated with the horseradish peroxidase-conjugated secondary antibody for 2 h at room temperature and washed again with TBST. Immunoreactivity was detected by the enhanced chemiluminescence detection reagents (KeyGen Biotechnology, Nanjing, China). The proteins were stripped and reblotted with anti-GAPDH antibody (Sigma) to verify the equal loading of protein in each lane.

### 2.10. Statistical Analysis

The statistical analyses were performed using one-way ANOVA with Tukey multiple comparison test by Graphpad 5.0. The results in the current study were presented as the means ± standard deviations (SDs). *P* value less than 0.05 presented statistical significance.

## 3. Results

### 3.1. Effects of Purslane on AST and ALT Activities in Serum and Liver Tissues

**Figure 1:**
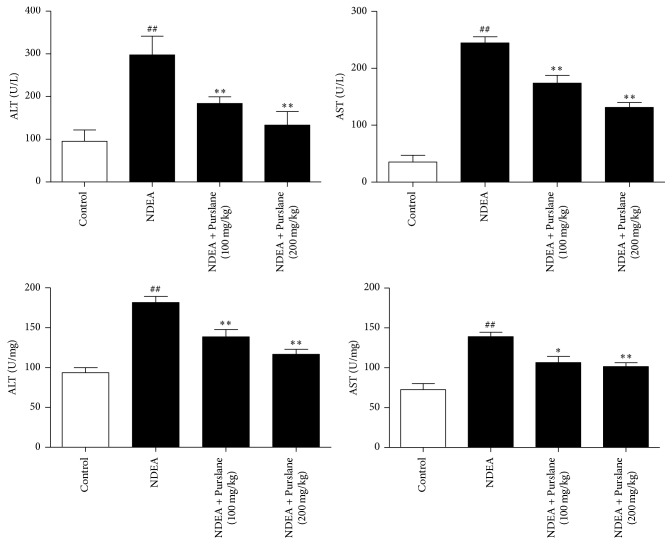
Effects of Purslane on AST and ALT activities in serum and liver tissues of NDEA-induced mice. The concentrations of ALT and AST in serum and liver tissues were determined using commercial kits according to the instructions recommended by the manufacturers. All values are expressed as means ± SDs. ^##^*P* < 0.01 versus control group. ^*∗*^*P* < 0.05 and ^*∗∗*^*P* < 0.01 versus NDEA group.

The AST and ALT activities were determined to assess liver injury. As illustrated in Figure 1, both the two transaminases in serum remained at low levels in control group. Notably, exposure to NDEA in mice significantly increased the activities of AST and ALT compared with those in control group, whereas treatment with Purslane (100 mg/kg and 200 mg/kg) effectively suppressed the serum levels of AST and ALT in NDEA-induced animals.

Moreover, NDEA stimulation also elevated the levels of AST and ALT in liver tissues. While the administration of Purslane (100 mg/kg and 200 mg/kg) significantly reduced the hepatic activities of AST and ALT, our data suggested that Purslane could ameliorate transaminase activity in liver cancer caused by NDEA challenge.

### 3.2. Effects of Purslane on Inflammatory Cytokines in Serum and in Liver

**Figure 2:**
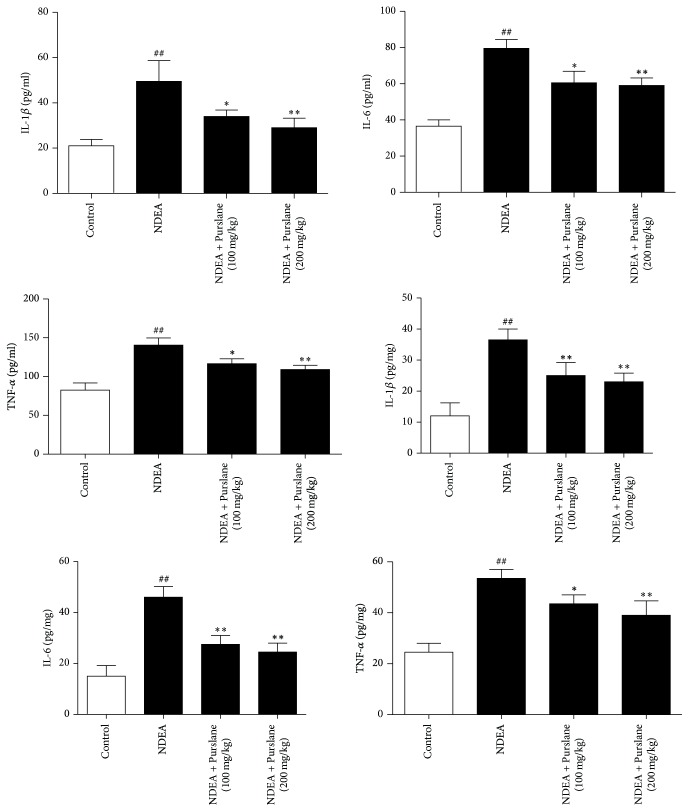
Effects of Purslane on the contents of inflammatory cytokines TNF-*α*, IL-6, and IL-1*β* in serum and liver of NDEA-induced mice. The serum and liver concentrations of TNF-*α*, IL-1*β*, and IL-6 were measured by ELISA kit in accordance with the manufacturers. The optical density (OD) was read at 450 nm with a microplate spectrophotometer. All values are expressed as means ± SDs. ^##^*P* < 0.01 versus control group. ^*∗*^*P* < 0.05 and ^*∗∗*^*P* < 0.01 versus NDEA group.

The effects of Purslane on serum and liver cytokines including TNF-*α*, IL-1*β*, and IL-6 were assayed with ELISA kits. As revealed in Figure 2, the levels of TNF-*α*, IL-1*β*, and IL-6 in the serum and liver samples presented apparent increases with NDEA stimulation as compared with those in control group. Purslane significantly inhibited the serum and liver contents of TNF-*α*, IL-1*β*, and IL-6 compared with those in the NDEA group.

### 3.3. Effects of Purslane on Lipid Peroxidation in Serum

**Figure 3:**
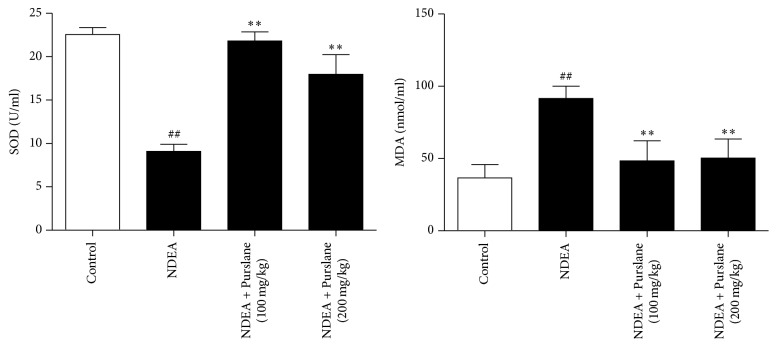
Effects of Purslane on the levels of SOD and MDA in serum of NDEA-induced mice. The operations for examination of SOD and MDA were conducted according to the instructions of commercial kits. All values are expressed as means ± SDs. ^##^*P* < 0.01 versus control group. ^*∗*^*P* < 0.05 and ^*∗∗*^*P* < 0.01 versus NDEA group.

MDA and SOD are known as biochemical markers of lipid peroxidation. As shown in Figure 3, drinking the water with NDEA evidently decreased the SOD level and increased the MDA content in serum. Nevertheless, treatment with Purslane (100 mg/kg and 200 mg/kg) remarkably restored the activity of SOD and reduced the content of MDA. The analytical data displayed that Purslane might alleviate the oxidative stress in NDEA-induced hepatic carcinoma.

### 3.4. Effect of Purslane on NDEA-Induced Pathological Changes of the Liver Tissues

**Figure 4:**
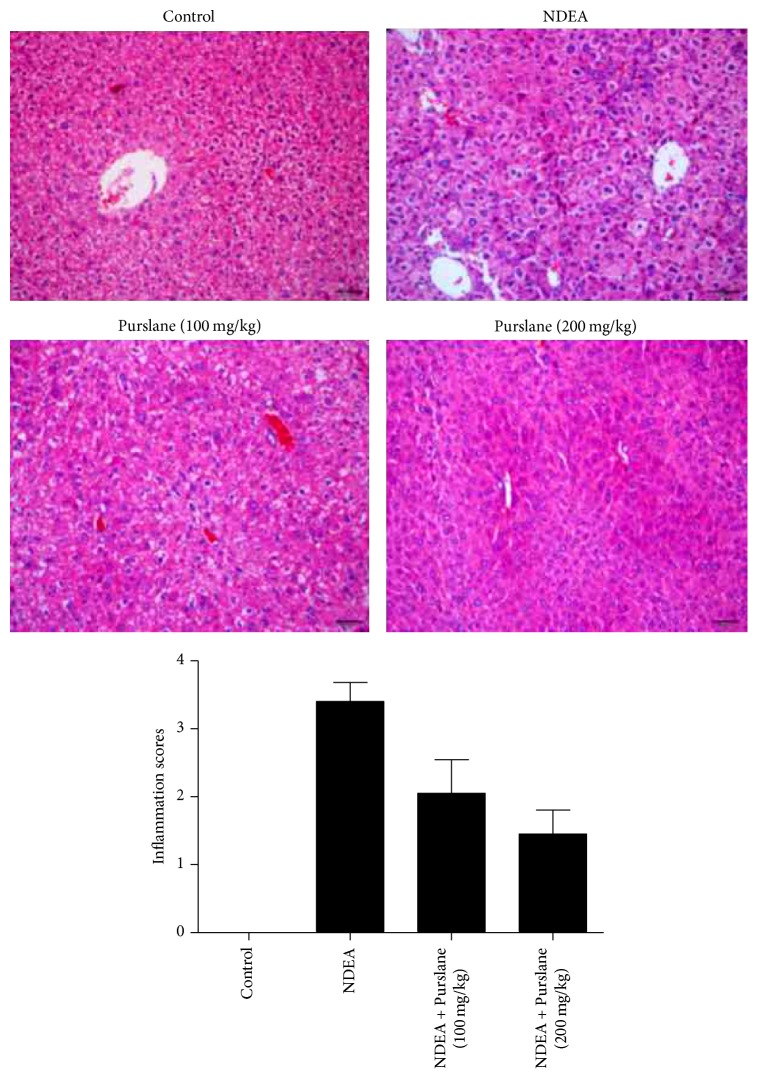
Effects of Purslane on pathological changes of the liver tissues of NDEA-induced mice. Liver inflammatory cell count based on a five-point scoring system was performed to estimate the severity of leukocyte infiltration. The scoring system was as follows: 0: no cells, 1: a few cells, 2: a ring of cells with a thickness of 1 cell layer, 3: a ring of cells with a thickness of 2–4 cell layers, and 4: a ring of cells with a thickness of more than 4 cell layers.

Hematoxylin and eosin (H&E) staining was conducted to observe the protective effect of Purslane on physiological impairment. As revealed in Figure 4, hepatic lobular architecture was clear and tightly aligned with large blue-hued nucleus that was seen in control mice, while the hepatic samples from NDEA-challenged mice presented nuclear fragmentation, cytoplasm condenses, fragmented desmosome complexes, and cell separated from whose neighbors. On the contrary, the severity of liver injury was attenuated by Purslane. Analytical results demonstrated that Purslane obviously ameliorated the histopathology condition in NDEA-induced liver cancer.

### 3.5. Effects of Purslane on NDEA-Induced PI3K/Akt/mTOR Pathway in Liver Tissues

**Figure 5:**
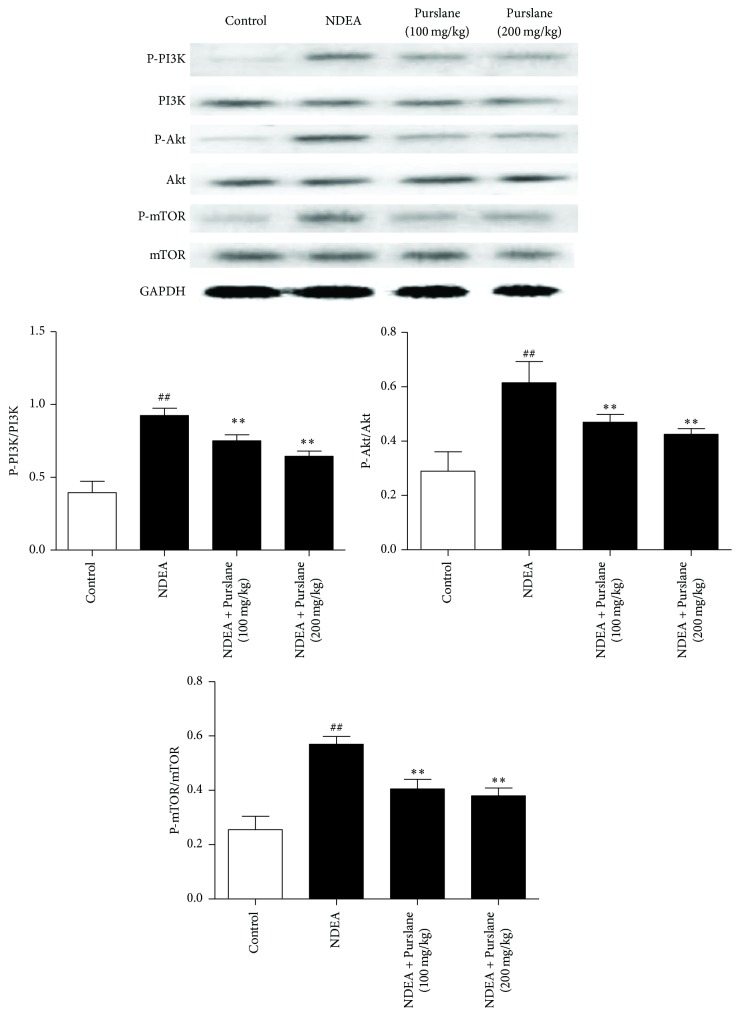
Effects of Purslane on the expressions of PI3K, Akt, and mTOR in liver tissues of NDEA-induced mice. All values are expressed as means ± SDs. ^##^*P* < 0.01 versus control group. ^*∗*^*P* < 0.05 and ^*∗∗*^*P* < 0.01 versus NDEA group.

To explore the hepatoprotective-associated signaling of Purslane treated mice, the phosphorylated and nonphosphorylated forms of the PI3K/Akt/mTOR pathway components were detected in liver tissues, respectively. As depicted in Figure 5, NDEA-injured mice showed obvious upregulated hepatic expressions of p-PI3K, p-Akt, and p-mTOR. However, Purslane effectively suppressed the phosphorylations of PI3K/Akt/mTOR signaling in liver tissues of NDEA-induced mice.

### 3.6. Effects of Purslane on the Protein Expressions of HO-1, Nrf2, and NF-*κ*B Signaling in NDEA-Induced Liver Tissues

Nrf2, HO-1, and NF-*κ*B signaling are the well-known molecular target controlling inflammation oxidative stress. In response to NDEA, both the protein levels of Nrf2 and HO-1 showed pronounced downregulations. In contrast, administration of Purslane significantly upregulated the expressions of Nrf2 and HO-1.

**Figure 6:**
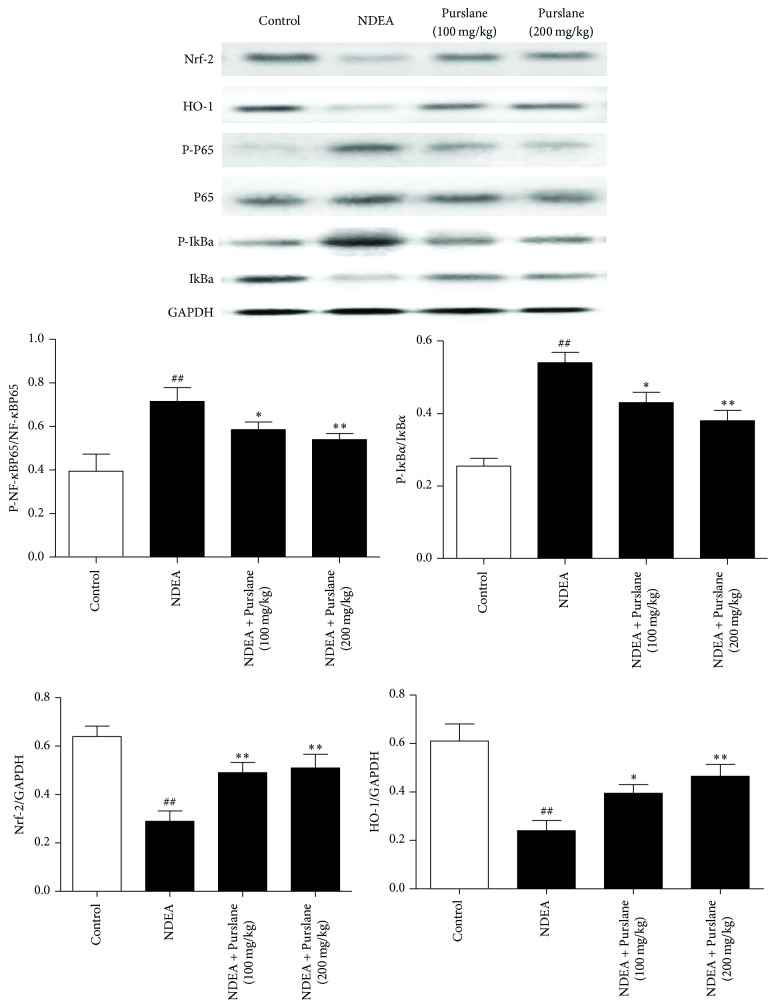
Effects of Purslane on the expressions of Nrf2 and HO-1 the phosphorylations of I*κ*B*α* and NF-*κ*B in liver tissues of NDEA-induced mice. All values are expressed as means ± SDs. ^##^*P* < 0.01 versus control group. ^*∗*^*P* < 0.05 and ^*∗∗*^*P* < 0.01 versus NDEA group.

Additionally, exposure to NDEA conduced to the upregulations of p-I*κ*B*α* and p-NF-*κ*Bp65, while treatment with Purslane dramatically blocked the phosphorylations of I*κ*B*α* and NF-*κ*Bp65. Our results suggested that the anti-inflammatory and antioxidative properties of Purslane might be attributed to the Nrf2/HO-1/NF-*κ*B pathway in NDEA-induced hepatic tumorigenesis (Figure 6).

## 4. Discussion

Liver cancer is common carcinomas with low survival rate. Since chemotherapeutic drugs are highly toxic and nonselective to normal cells, the development of more effective and safety compounds from natural product is a relatively significant strategy for cancer therapy. NDEA has been used by different investigators to induce liver tumors in animals. In response to NDEA stimulation, cytoplasmic transaminase including ALT and AST is released and enters circulation. Both ALT and AST serve as reliable diagnostic indicators of liver injury [19]. The data displayed that the activities of transaminases significantly increased, which confirmed the successful building of NDEA-induced HCC model. Whereas the Purslane inhibited the levels of ALT and AST, which indicated its hepatoprotective effects. The pathological observation also further proved that Purslane could ameliorate the liver cancer.

Several investigators pointed out the pivotal role of inflammatory modulation in the progression of hepatocellular carcinomas [20]. The inflammatory cytokines including TNF-*α*, IL-6, and IL-1*β* were overproduced in hepatic injury [21]. The data of the current study demonstrated that Purslane markedly inhibited the generations of inflammatory cytokines, which suggested the anti-inflammatory activity of Purslane in NDEA-induced mice.

Excessive free radicals produced during the process of NDEA metabolism in liver tissue contribute to the disruption of chromosomal integrity and DNA damage. Previous evidence found NDEA exposure results in high MDA formation [22]. Lipid peroxidation is one of most extensively studied manifestations of oxygen toxicity [23]. As a byproduct of lipid peroxidation, MDA is generated under oxidative stress and considered as reliable index for oxidative damage which attributed to the plasma membrane and the resultant production [24]. SOD is the critical enzyme governing redox balance and scavenging superoxide radicals [25]. Former research confirmed the involvements of SOD and MDA in the development of liver tumorigenesis [26]. Our experimental data proved that Purslane exerted antioxidative effect on NDEA-induced mice.

PI3K/Akt signaling is the upstream molecule driving mechanistic target of mTOR. In mammals, mTOR is mediated by a kinase cascade involving Akt molecule which is governed by PI3K [27]. mTOR belongs to the PI3K related kinase family and is closely associated with the regulation of cell viability and proliferation [28]. The positive regulation of mTOR by PI3K/Akt signaling pathway was found to be involved in the pathogenesis of cancer [29]. PI3K/Akt/mTOR also participates in the process of hepatocellular carcinoma [30]. The results of western blot demonstrated that administration of Purslane effectively inhibited the phosphorylations of PI3K/Akt/mTOR pathway in NDEA-challenged mice.

To adapt to excessive reactive oxygen species (ROS) and subsequent lipid peroxidation, cancer cells activate Nrf2 pathway, an important pathway regulating oxidative stress. It is proposed that the inhibition of the PI3K/AKT/mTOR axis is redox-dependent [31]. Notably, application of NAC (N-acetyl-cysteine, a ROS scavenger) alone has been proved to suppress mTOR signaling. ROS functioning as a signaling molecule plays both activating and inhibitory roles in mTOR signaling [32]. High level of ROS may give rise to the disruption of Nrf2/Kelch-like ECH associated protein-1 (Keap1) complex and thus results in the translocation of Nrf2 from cytoplasm to nucleus [33]. Thereafter, Nrf2 combines with antioxidant response element (ARE) to activate the downstream targets HO-1 which eventually promote antioxidant system [34]. Interestingly, HO-1 can manifest both antioxidant and anti-inflammatory activities [35]. Furthermore, former literature raised the relationship between Nrf2/HO-1 and NF-*κ*B signaling [36]. NF-*κ*B is the essential event accounting for the transcriptions of inflammatory cytokines TNF-*α*, IL-6, and IL-1*β* [37]. NF-*κ*B is activated by the phosphorylation and degradation of I*κ*B*α* [38]. It has also been proposed that mTOR could regulate NF-*κ*B in feedback or directly [39]. Numerous evidence confirmed the involvement of Nrf2/HO-1/NF-*κ*B in the development of hepatic tumorigenesis [40, 41]. Our experimental results indicated that Purslane was capable of upregulating the expressions of Nrf2 and HO-1 and blocked the phosphorylations of I*κ*B*α* and NF-*κ*Bp65 in NDEA-stimulated mice.

In conclusion, the present study demonstrated that Purslane exhibited protective effect on NDEA-induced hepatocellular carcinomas by inhibiting inflammatory response and oxidative stress possibly through the PI3K/AKT/mTOR and Nrf2/HO-1/NF-*κ*B pathway. Further researches are warranted before clinical application of Purslane.
